# Cognitive Stimulation Therapy in Outpatient Settings for Persons with Dementia

**DOI:** 10.1093/geroni/igab046.3371

**Published:** 2021-12-17

**Authors:** Max Zubatsky, Marla Berg-Weger, Matthew Amick, Debbie Blessing, Katelyn Sansone, Anna Brende, Janice lundy

**Affiliations:** 1 Medical Family Therapy Program, Saint Louis University, Missouri, United States; 2 Saint Louis University, Saint Louis, Missouri, United States; 3 Saint Louis University, Saint Louis University, Missouri, United States; 4 A.T. Still University of Health Sciences, A.T. Still University, Missouri, United States; 5 Perry County Memorial Hospital, Perry County Memorial Hospital, Missouri, United States

## Abstract

Cognitive Stimulation Therapy (CST) is a non-pharmacologic evidence-based intervention for persons living with mild to moderate dementia. This clinical intervention therapy follows a structured protocol designed to connect people with memory loss to others by providing opportunities for social engagement and group discussion of current events and a different themed activity each session. This presentation will include findings from a multi-site study of the group intervention conducted in an urban and two rural out-patient settings with community-dwelling older adults. Pre- and post-assessments captured data on cognitive function, depression, quality-of-life, and mobility. While CST is offered in over 30 countries, this is the first large-scale CST study conducted in the U.S. Implications for future practice and research will be presented.

